# Advancing Clinical Research

**DOI:** 10.1002/advs.74159

**Published:** 2026-02-03

Antonia Eisenkoeck, John Wiley & Sons Ltd., aeisenkoec@wiley.com

Ada Hang‐Heng Wong, John Wiley & Sons Ltd., adwong@wiley.com

Since its inception, *Advanced Science* has championed cutting‐edge, cross‐disciplinary research, striving to bridge foundational discovery and real‐world impact. In recent years, we have witnessed, welcomed, and intentionally introduced clinical research into this journal. In response, we now step forward more proactively to establish *Advanced Science* as a rigorous and supportive home for clinical research at the highest level.

Upholding integrity and global best practices, we have refined and expanded our editorial policies to support our clinical community. These updates codify expectations around ethical approvals, prospective trial registration, patient consent, data‐sharing, and transparent reporting. By aligning with international standards such as the International Committee of Medical Journal Editors (ICMJE) and the World Health Organization (WHO), we reinforce our commitment to research integrity, reproducibility, and trust.

For clinical trials, registration preceding study initiation is verified. Where appropriate, we review the submitted protocols (including amendments) and share them with peer reviewers. We expect adherence to CONSORT reporting standards, and discourage selective presentation of outcomes and ad hoc analyses without clear disclosure of their status.

Moreover, we encourage early protocol registration at our sister journal Current Protocols to promote transparency and reproducibility, and also invite community feedback before data collection. In addition, we encourage deposition of clinical data in public data repositories. In cases where privacy or regulatory constraints prevent full data release, a data‐sharing plan needs to be included in the submission. These measures help ensure that clinical studies published in *Advanced Science* are ethically sound, methodologically robust, and transparently reported.

Demonstrating our commitment to clinical science, we have curated a collection of recently published clinical studies to showcase the breadth of clinical research already featured in *Advanced Science*. Together, these studies illustrate how precision medicine, clinical innovation, and enabling technologies can transform patient care. For example, Qi et al. introduced a gene therapy approach to restore hearing in autosomal recessive deafness 9, while Fu et al. validated oxytocin's role in reducing fear responses via middle cingulate–amygdala pathways. The TAPAS trial proposed adaptive physical activity to enhance stroke rehabilitation, and the VitaDEx trial showed that moderate exercise supports vitamin D homeostasis in overweight adults. Xiao et al. identified an inflection point predicting long‐term outcomes in hepatitis B virus–related acute‐on‐chronic liver failure, and Wu et al. combined circulating tumor DNA with 18F‑fludeoxyglucose positron emission tomography as a less invasive alternative for forecasting chemoradiotherapy response in non–small cell lung cancer. We hope this collection inspires clinicians and translational researchers to share their most ambitious studies with us.

Strengthening our foothold in clinical research, Wiley Advanced proudly partners with MD Anderson Cancer Center in hosting the forthcoming 2026 International Cancer Neuroscience Symposium. This meeting will convene leading experts to explore emerging interfaces between cancer biology and neural diseases, fostering global collaboration and discourse at the frontiers of clinical translation.

As clinical science continues to advance, *Advanced Science* stands ready as a rigorous, open, and trusted platform for bold clinical studies. Clinicians, translational researchers, and multidisciplinary teams are invited to submit their most ambitious work. Together, we drive progress in transparent, reproducible, and high‐impact clinical research, for the benefit of patients worldwide.

The *Advanced Science* Editorial Team

